# Supplementary material for: Efficacy and Safety of Plasma Exchange as an Adjunctive Therapy for Rapidly Progressive IgA Nephropathy and Henoch-Schönlein Purpura Nephritis: A Systematic Review
Source: Int J Mol Sci. 2023 Feb 16;24(4):3977. doi: 10.3390/ijms24043977 (PMC9958587; doi:10.3390/ijms24043977)
Supplement: Supplementary file 1 [file ijms-24-03977-s001.zip › ijms-2185037-supplementary.pdf]

	Does the patient represent the whole experience of the investigators/center?	Was the exposure adequately ascertained?	Was the outcome adequately ascertained?	Were there alternative causes that may explain the observation ruled out?	Was there a challenge/rechallenge phenomenon?	Was there a dose-response effect?	Was follow up long enough for outcomes to occur?	Is the case described with sufficient details to allow other investigators to replicate the research?
**Coppo 1985**	Yes	Yes	Yes	No	n/a	n/a	Yes	Yes
**Tejeiro 1990**	No	Yes	Yes	Yes	n/a	n/a	Yes	Yes
**Streather 1994**	No	Yes	Yes	Yes	n/a	n/a	Yes	Yes
**Affessa 1997**	Yes	Yes	Yes	Yes	n/a	n/a	Yes	Yes
**McGregor 1998**	Yes	Yes	Yes	Yes	n/a	n/a	Yes	Yes
**Chen 2004**	Yes	Yes	Yes	Yes	n/a	n/a	Yes	Yes
**Rech 2005**	Yes	Yes	Yes	Yes	n/a	n/a	Yes	Yes
**Fujinaga 2006**	Yes	Yes	Yes	Yes	n/a	n/a	Yes	Yes
**Anatham 2007**	Yes	Yes	Yes	Yes	n/a	n/a	Yes	Yes
**Wang 2011**	Yes	Yes	Yes	Yes	n/a	n/a	Yes	Yes
**Pipilli 2012**	Yes	Yes	Yes	Yes	n/a	n/a	Yes	Yes
**Herzog 2014**	Yes	Yes	Yes	Yes	n/a	n/a	Yes	Yes
**Otsuka 2014**	Yes	Yes	Yes	Yes	n/a	n/a	No	No
**Yim 2014**	Yes	Yes	Yes	Yes	n/a	n/a	Yes	No
**Hamilton 2015**	Yes	Yes	Yes	Yes	n/a	n/a	Yes	Yes
**Ring 2015**	Yes	Yes	Yes	Yes	n/a	n/a	Yes	Yes
**Doddi 2016**	Yes	Yes	Yes	Yes	n/a	n/a	No	Yes
**Pannu 2016**	Yes	Yes	Yes	No	n/a	n/a	No	No
**Nissaisorakarn 2017**	No	Yes	Yes	Yes	n/a	n/a	Yes	Yes
**Soltanpour 2017**	No	Yes	Yes	No	n/a	n/a	No	No
**Vega 2017**	Yes	Yes	Yes	Yes	n/a	n/a	No	No
**Surmelidoven 2018**	Yes	Yes	Yes	Yes	n/a	n/a	Yes	Yes
**Rajiv 2018**	Yes	Yes	Yes	No	n/a	n/a	No	Yes
**Gani 2019**	Yes	Yes	Yes	Yes	n/a	n/a	Yes	Yes
**Kojima 2019**	Yes	Yes	Yes	Yes	n/a	n/a	Yes	Yes
**Longano 2019**	Yes	Yes	Yes	Yes	n/a	n/a	Yes	Yes
**Bhuwania 2020**	No	Yes	Yes	Yes	n/a	n/a	Yes	Yes
**Apaydin 2021**	Yes	Yes	Yes	Yes	n/a	n/a	Yes	Yes
**Zhang 2021**	Yes	Yes	Yes	Yes	n/a	n/a	Yes	Yes

Supplemental Table S1: Methodological quality of included case reports. Tool adapted from *Methodological quality and synthesis of case series and case reports published* in 2018 by Murad et al. (n/a, not applicable)

	Does the patient(s) represent the whole experience of the investigators/center?	Was the exposure adequately ascertained?	Was the outcome adequately ascertained?	Were there alternative causes that may explain the observation ruled out?	Was there a challenge/rechallenge phenomenon?	Was there a dose-response effect?	Was follow up long enough for outcomes to occur?	Is the case described with sufficient details to allow other investigators to replicate the research?
**Lai 1987**	Yes	Yes	Yes	No	n/a	n/a	Yes	Yes
**Nicholls 1990**	No	Yes	Yes	No	n/a	n/a	Yes	Yes
**Rocatello 1995**	No	Yes	Yes	No	n/a	n/a	Yes	Yes
**Gianviti 1996**	Yes	Yes	Yes	No	n/a	n/a	Yes	Yes
**Shenoy 2007**	Yes	Yes	Yes	No	n/a	n/a	Yes	Yes
**Wright 2006**	Yes	Yes	Yes	Yes	n/a	n/a	Yes	Yes
**Xie 2016**	Yes	Yes	Yes	Yes	n/a	n/a	Yes	Yes
**Chambers 1999**	No	Yes	Yes	No	n/a	n/a	No	Yes
**Rajgopala 2017**	No	Yes	Yes	No	n/a	n/a	Yes	Yes

Supplemental Table S2: Methodological quality of included case series. Tool adapted from *Methodological quality and synthesis of case series and case reports* published in 2018 by Murad et al. (n/a, not applicable)

Murad MH, Sultan S, Haffar S, Bazerbachi F. Methodological quality and synthesis of case series and case reports. *BMJ Evid Based Med*. 2018;23(2):60-63. doi:10.1136/bmjebm-2017-110853
